# Impact of Structured Preoperative Counseling on Anaesthesia Preference in Elective Cesarean Delivery: A Prospective Study

**DOI:** 10.4274/TJAR.2026.262616

**Published:** 2026-10-30

**Authors:** Seval Kılbasanlı, Osman Aydın, Bülent Çakmak

**Affiliations:** 1 Niğde Ömer Halisdemir University Faculty of Medicine, Department of Anaesthesiology and Reanimation, Niğde, Türkiye; 2 Maltepe Ersoy Hospital, Clinic of Obstetrics and Gynecology, İstanbul, Türkiye; 3 Kurtköy Ersoy Hospital, Clinic of Obstetrics and Gynecology, İstanbul, Türkiye

**Keywords:** Cesarean delivery, spinal anaesthesia, general anaesthesia, patient preference, patient education, decision making

## Abstract

**Objective:**

To assess changes in anaesthesia preference before and after structured preoperative counseling and to identify factors associated with anaesthesia preference among women scheduled for elective cesarean delivery.

**Methods:**

This prospective, single-center, single-arm, pre-post observational study included 200 pregnant women classified as the American Society of Anesthesiologists physical status II. Data were collected using a structured questionnaire assessing demographic characteristics, obstetric history, previous anaesthesia experience, level of knowledge, and anaesthesia preference, both before and after counseling. Statistical analyses were performed using SPSS version 22.0, and a* P* value < 0.05 was considered statistically significant.

**Results:**

At baseline, 53% of patients preferred spinal anaesthesia and 47% preferred general anaesthesia. Following counseling, the preference for spinal anaesthesia increased to 88.5% (*P* < 0.001). Increasing age was associated with a decreased likelihood of choosing spinal anaesthesia [odds ratio (OR)=0.94, 95% confidence interval (CI): 0.89-0.99], whereas higher education (OR=1.78, 95% CI: 1.05-3.02), prior knowledge (OR=1.65, 95% CI: 1.02-2.68), and previous spinal anaesthesia experience (OR=2.95, 95% CI: 1.65-5.28) were identified as independent predictors of spinal anaesthesia preference. No significant association was found between smoking and anaesthesia preference, or between comorbidities and anaesthesia preference. The postoperative satisfaction rate with the anaesthesia was 91.5%.

**Conclusion:**

Following structured preoperative counseling, the preference for neuraxial anaesthesia increased among women scheduled for elective cesarean delivery. These findings suggest that standardized patient education may be a useful component of preoperative anaesthesia assessment.

**Main Points**

• Anaesthesia preference for elective cesarean delivery was associated with patient-related factors, including education level, prior knowledge, and experience with spinal anaesthesia.

• Preference for neuraxial (spinal) anaesthesia increased following structured preoperative counseling.

• Fear-related concerns, such as paralysis and intraoperative pain, play a major role in the choice of general anaesthesia.

• Higher educational level and prior knowledge of anaesthesia are associated with increased preference for spinal anaesthesia.

• Structured preoperative information may support patient-centered discussions during preoperative anaesthesia assessment.

## Introduction

Cesarean delivery is one of the most commonly performed surgical procedures worldwide, and anaesthesia management plays a critical role in both maternal and neonatal outcomes. While neuraxial anaesthesia is currently recommended as the preferred approach for cesarean delivery, general anaesthesia is still used in certain situations depending on clinical conditions, urgency, maternal comorbidities, and patient preference.^1, 2^ Neuraxial anaesthesia offers several advantages, including improved postoperative analgesia, preservation of maternal awareness, and a lower risk of neonatal depression.^3, 4^

The choice of anaesthesia technique in cesarean delivery is influenced not only by clinical requirements but also by its impact on maternal and neonatal outcomes. Although neuraxial techniques are generally associated with better neonatal outcomes, general anaesthesia remains essential in emergency situations due to its rapid applicability.^5, 6^ In addition, some studies suggest that general anaesthesia may be associated with adverse psychological outcomes, including postpartum depression.^7^ In high-risk obstetric conditions such as placenta previa and placenta accreta spectrum, the choice of anaesthesia becomes more complex and requires an individualized approach.^8, 9^

With the increasing emphasis on patient-centered care, patients’ knowledge, expectations, and anxiety levels have been recognized as key determinants of anaesthesia preference. Previous studies have shown that appropriate preoperative information can reduce anxiety and increase the preference for neuraxial anaesthesia.^10^ However, patients’ and healthcare providers’ priorities may differ, which can influence decision-making processes.^11, 12^ In particular, misconceptions and fear-related concerns remain important factors driving the preference for general anaesthesia.^13^ These concerns may include fear of paralysis, intraoperative pain, awareness, back pain, and post-dural puncture headache; structured counseling may help address these misconceptions more systematically than routine, unstructured preoperative information.

Although previous studies have shown that preoperative education may influence anaesthesia preference, most have focused on comparing different educational methods or evaluating anxiety reduction. Evidence regarding the integration of standardized, structured face-to-face counseling into routine anaesthesiology practice and its association with changes in anaesthesia preference remains limited. Furthermore, studies evaluating both pre- and post-counseling anaesthesia preferences, together with patient-related factors influencing these preferences, are scarce. Therefore, this study aimed to assess changes in anaesthesia preference before and after structured preoperative counseling and to identify factors associated with anaesthesia preference among women scheduled for elective cesarean delivery.

## Methods

This prospective, single-center, single-arm, pre-post observational study was conducted in pregnant women scheduled for elective cesarean delivery. The study was designed to evaluate changes in anaesthesia preference before and after (Supplementary Material 1) structured preoperative counseling. Due to the absence of a control group, the study design allowed the assessment of changes in preference following counseling, but did not allow causal inference regarding the effect of counseling.

The study was approved by the Niğde Ömer Halisdemir University, Non-Interventional Clinical Research Ethics Committee (approval no.: 2022/32, date: April 23, 2022) and conducted in accordance with the Declaration of Helsinki. Written informed consent was obtained from all participants.

The sample size calculation was based on an anticipated absolute increase of 20% in spinal anaesthesia preference following preoperative counseling. This conservative effect size was selected based on the findings of Karataş et al.,^10^ who reported an increase in spinal anaesthesia preference from 36% before preoperative information to 73% after educational intervention among women scheduled for elective cesarean delivery. To avoid overestimating the expected effect and to ensure an adequate sample size, a more conservative absolute increase of 20% was used for the power analysis. To achieve 80% power with a significance level of α = 0.05, a minimum of 185 participants was required. Accounting for a 10% anticipated dropout rate due to incomplete data or emergency status, 200 pregnant women were included in the study.

Pregnant women aged 18 years or older, classified as American Society of Anesthesiologists physical status II and scheduled for elective cesarean delivery, who agreed to participate and were able to complete the study questionnaire, were eligible for inclusion. Patients undergoing emergency cesarean delivery, those who declined to participate, those unable to complete the questionnaire, and those with incomplete data were excluded.

Data were collected using a standardized questionnaire assessing demographic variables, obstetric history, prior anaesthesia experiences, anaesthesia preferences, reasons for preference, and patient satisfaction. The questionnaire used in the study is provided as Supplementary Material 2. Participants self-administered a questionnaire specifically developed by the investigators for this study. No formal validation study was performed. Patients’ initial anaesthesia preferences (spinal or general) were recorded. Subsequently, all participants received a standardized 15-minute face-to-face preoperative counseling session from an experienced anaesthesiologist. This session included information on the technical aspects of spinal and general anaesthesia, on their clinical advantages and potential risks, and targeted clarification of common misconceptions such as the risk of paralysis or intraoperative awareness. The counseling was delivered using a structured verbal format with predefined key components to ensure consistency across participants. All sessions were conducted by the same experienced anaesthesiologist, and no written materials or visual aids were used.

The counseling session was conducted according to a predefined, structured counseling form developed by the investigators. The counseling covered available anaesthesia options for cesarean delivery, the procedure and characteristics of spinal anaesthesia, advantages and disadvantages of spinal and general anaesthesia, common concerns and misconceptions (including paralysis, intraoperative pain, awareness, back pain, and post-dural puncture headache), maternal-infant interaction, early breastfeeding, and postoperative pain management strategies. Patients were encouraged to ask questions throughout the session. To ensure consistency, all counseling sessions were delivered by the same experienced anaesthesiologist using the same structured framework.

Following this structured session, anaesthesia preferences were reassessed to evaluate changes resulting from counseling. The primary outcome was the change in anaesthesia preference, while secondary outcomes included patient satisfaction and specific reasons for anaesthesia choice. A change in anaesthesia preference was defined as a shift in the participant’s preferred anaesthesia technique (spinal or general anaesthesia) between the pre-counseling and post-counseling assessments. Patient satisfaction was assessed at 24 hours postoperatively using a binary (yes/no) question about satisfaction with the anaesthesia received. No validated satisfaction scale was used. Data on the anaesthesia technique administered and any conversions from spinal to general anaesthesia were also recorded in the anaesthesia records.

### Statistical Analysis

Statistical analyses were performed using SPSS version 22.0 (IBM Corp., Armonk, NY, USA). Normality of the data distribution was assessed using appropriate tests. Continuous variables were expressed as mean ± standard deviation or median (minimum-maximum), depending on data distribution. Categorical variables were presented as numbers and percentages. Group comparisons were performed using the chi-square test or Fisher’s exact test for categorical variables and the Student’s t-test or Mann-Whitney U test for continuous variables, as appropriate. The McNemar test was used to evaluate changes in anaesthesia preference before and after counseling.

Multivariable logistic regression analysis was performed to identify factors independently associated with baseline preference for spinal anaesthesia. The dependent variable was the initial anaesthesia preference (spinal anaesthesia vs. general anaesthesia). Variables demonstrating statistical significance in univariate analyses, together with clinically relevant variables, were entered into the multivariable logistic regression model. Education level was categorized as higher (high school or university) or lower (no schooling or primary school). Collinearity diagnostics were performed using variance inflation factor (VIF) analysis, and no evidence of multicollinearity was identified (all VIF values <2). A *P *value < 0.05 was considered statistically significant.

## Results

A total of 200 pregnant women were included in the study. The demographic and obstetric characteristics of the participants are presented in Table 1.

Overall, anaesthesia preferences are summarized in Table 2. At baseline, 106 women (53.0%) preferred spinal anaesthesia and 94 (47.0%) preferred general anaesthesia. Following structured preoperative counseling, the number of women preferring spinal anaesthesia increased to 177 (88.5%), while those preferring general anaesthesia decreased to 23 (11.5%). Specifically, 82 women (87.2%) changed their preference from general to spinal anaesthesia, whereas 11 women (10.4%) changed from spinal to general anaesthesia (McNemar test, *P* < 0.001). The preferred anaesthesia technique after counseling was initially attempted in all participants. Spinal anaesthesia was initiated in 177 patients and general anaesthesia in 23 patients. Among patients in whom spinal anaesthesia was initiated, 4 required conversion to general anaesthesia. Therefore, the final anaesthesia techniques used were spinal anaesthesia in 173 patients and general anaesthesia in 27 patients. Overall concordance between the post-counseling preference and the final anaesthesia technique received was 98.0%. Patient satisfaction was assessed 24 hours postoperatively using a binary yes/no question regarding satisfaction with the anaesthesia received. Overall, 183 patients (91.5%) reported satisfaction with the anaesthesia received. Among the 4 patients who required conversion from spinal to general anaesthesia, 3 were satisfied with the anaesthesia received. The participant flow and anaesthesia preferences before and after counseling are illustrated in Figure 1.

Patients who initially preferred spinal anaesthesia were significantly younger than those who preferred general anaesthesia. In addition, a higher level of education, prior information about anaesthesia, and previous experience with spinal anaesthesia were significantly associated with preference for spinal anaesthesia. No significant differences were observed between the groups in terms of gravida, parity, gestational age, smoking status, or comorbidities (Table 3).

The reasons for anaesthesia preference differed between the groups. The desire to see the baby immediately (45%) and early breastfeeding (38%) were the most frequently reported reasons for preferring spinal anaesthesia, whereas fear-related concerns—particularly fear of paralysis (44%), pain, and post-procedural headache—were more common among patients who preferred general anaesthesia.

In multivariable logistic regression analysis, increasing age was associated with a decreased likelihood of choosing spinal anaesthesia. In contrast, higher educational level, prior information about anaesthesia, and previous experience with spinal anaesthesia were independently associated with an increased preference for spinal anaesthesia. Smoking status and comorbidities were not significantly associated with anaesthesia preference (Table 4).

## Discussion

In this study, the factors influencing anaesthesia preference in pregnant women scheduled for elective cesarean delivery were evaluated from multiple perspectives. Our findings demonstrate that anaesthesia preference is influenced not only by clinical necessity but also by patient-related factors, including education level, prior knowledge, and experience with spinal anaesthesia. These findings highlight the importance of patient-centered approaches in anaesthesia decision-making.

Neuraxial anaesthesia is widely recommended as the standard approach for cesarean delivery due to its well-established benefits for both maternal and neonatal outcomes.^1, 2^ Despite these advantages, nearly half of the participants in our study initially preferred general anaesthesia, indicating a discrepancy between clinical recommendations and patient perceptions. This finding is consistent with previous studies emphasizing the role of patient knowledge and perception in shaping anaesthesia choice.^10^

One of the key findings of this study is the substantial increase in the preference for spinal anaesthesia following structured preoperative counseling. This finding indicates that anaesthesia preferences may change following structured preoperative counseling. However, due to the observational pre-post design and the absence of a control group, the observed change cannot be attributed solely to the counseling intervention. Previous studies have similarly reported that appropriate preoperative information reduces anxiety and increases the preference for neuraxial anaesthesia.^10^ The magnitude of change observed in our study appears higher than that reported in previous studies, which may be attributable to differences in baseline patient knowledge and the structured counseling provided. In addition, the post-counseling preferred anaesthesia technique was initially attempted for all participants, and concordance between post-counseling preference and the final anaesthesia technique was high. However, the concordance finding should be interpreted with caution because the study was not designed to evaluate validated patient-centered outcomes or the detailed reasons for conversion.

The analysis of the reasons for anaesthesia preference indicates that patient decisions are strongly influenced by perceptual and emotional factors. While the desire to see the baby immediately and to initiate early breastfeeding were the main drivers of preference for spinal anaesthesia, fear-related concerns were more prominent among patients preferring general anaesthesia. In particular, fears such as paralysis, intraoperative pain, and headache appear to play a decisive role, although they are not always supported by clinical evidence.^13^ These findings underline the importance of addressing misconceptions and providing accurate, targeted information during preoperative counseling.

Multivariable analysis demonstrated that a higher level of education, prior knowledge about anaesthesia, and previous experience with spinal anaesthesia were independent predictors of preference for spinal anaesthesia. These results suggest that health literacy and prior exposure play a critical role in shaping patient choices.^10, 11^ In contrast, older age was associated with a lower likelihood of preferring spinal anaesthesia, which may reflect greater anxiety or more established beliefs among older patients.

No significant association was found among smoking, comorbidities, and anaesthesia preference. Although these factors are clinically relevant to obstetric outcomes, their limited influence on choice of anaesthesia suggests that cognitive and experiential factors may play a more decisive role in this context.

### Study Limitations

This study has several limitations. First, it was conducted at a single center using a single-arm, pre-post observational design without a control group; therefore, causal relationships between counseling and changes in anaesthesia preference cannot be established. Second, having all counseling sessions delivered by the same anaesthesiologist may have introduced interviewer bias, physician authority bias, and social desirability bias. Third, the questionnaire used in the study was not a formally validated instrument. Fourth, patient satisfaction was assessed using a simple binary question rather than a validated satisfaction scale. Because of the small number of patients requiring conversion from spinal to general anaesthesia and the binary nature of the satisfaction assessment, no formal subgroup statistical comparison of satisfaction by conversion status was performed. Although the actual anaesthesia technique administered and any conversions from spinal to general anaesthesia were recorded, detailed clinical reasons for conversion and validated patient-centered outcomes, such as decisional conflict, anxiety reduction, and quality of decision-making, were not evaluated. These limitations should be considered when interpreting the findings.

## Conclusion

Anaesthesia preference during elective cesarean delivery was associated with patient-related factors, including educational level, prior knowledge of anaesthesia, and prior experience with spinal anaesthesia. Preference for neuraxial anaesthesia increased following structured preoperative counseling. In addition, higher education level, prior information about anaesthesia, and previous spinal anaesthesia experience were independently associated with a greater likelihood of preferring spinal anaesthesia. These findings suggest that standardized patient education may be a useful component of routine preoperative anaesthesia assessment.

## Ethics

**Ethics Committee Approval: **The study was approved by the Niğde Ömer Halisdemir University, Non-Interventional Clinical Research Ethics Committee (approval no.: 2022/32, date: April 23, 2022) and conducted in accordance with the Declaration of Helsinki.

**Informed Consent: **Written informed consent was obtained from all participants.

## Supplementary Materials

Supplementary Material 1 https://d2v96fxpocvxx.cloudfront.net/72b4fafb-5db4-4d61-bedf-c8100360bb15/content-images/51fed6f1-2910-4176-81c6-a2e6b434c615.pdf

Supplementary Material 2 https://d2v96fxpocvxx.cloudfront.net/72b4fafb-5db4-4d61-bedf-c8100360bb15/content-images/83b7bc5a-1312-4cd6-8afd-0555cbb05367.pdf

## Figures and Tables

**Figure 1:**
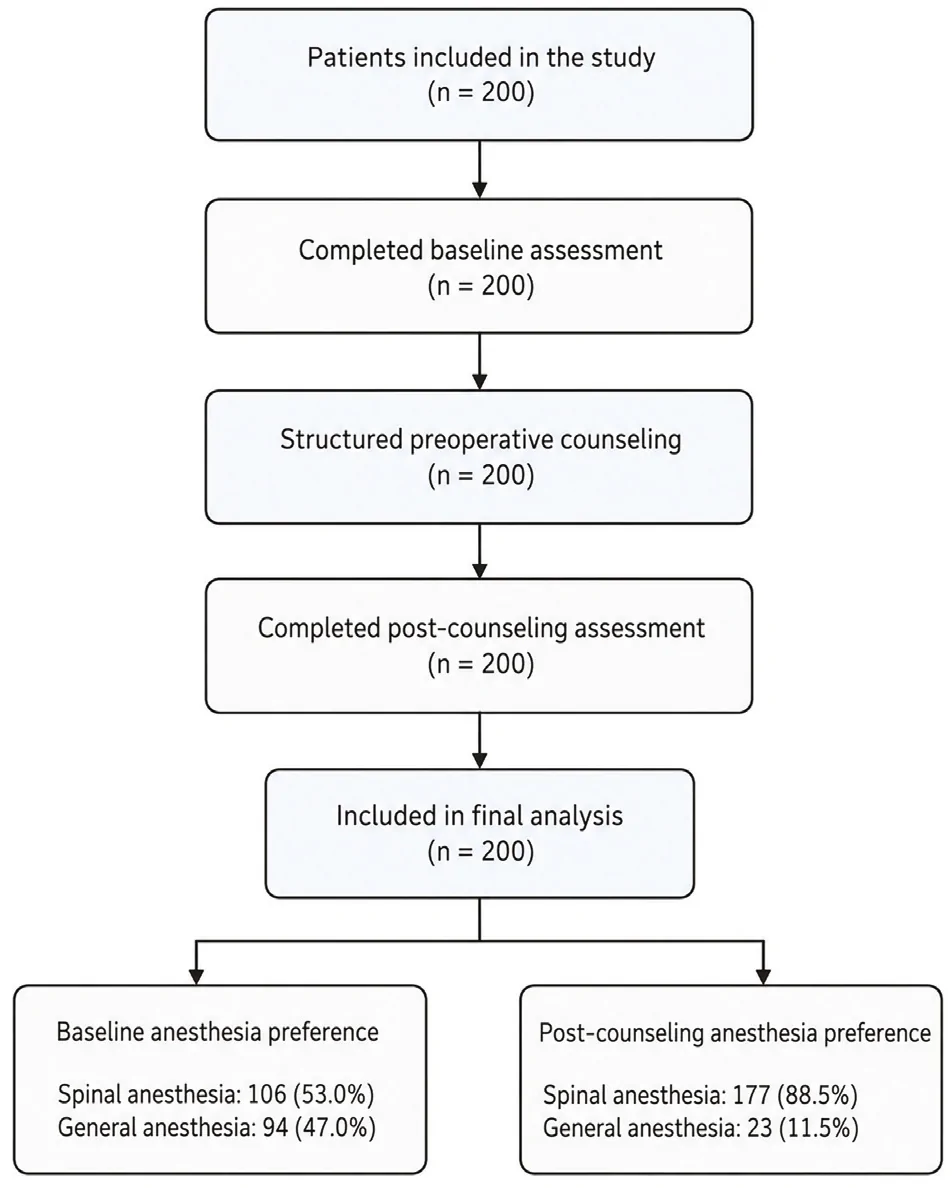
Participant flow diagram illustrating patient inclusion, completion of baseline and post-counseling assessments, structured preoperative counseling, and anaesthesia preference before and after counseling.

**Table 1. Demographic and Obstetric Characteristics (n = 200):** 

**Variable**	**Value**
Age (years)	27.4±3.9
Height (cm)	162.1±5.1
Weight (kg)	68.7±8.8
Gestational age (weeks)	38.0±0.8
Gravida	2.3±1.0
Parity	1.1±0.8
Comorbidity (yes)	17.0% (n = 34)
Smoking (yes)	13.0% (n = 26)
Education-no schooling	9.0% (n = 18)
Education-primary school	36.5% (n = 73)
Education-high school	32.5% (n = 65)
Education-university	22.0% (n = 44)
Occupation-housewife	61.0% (n = 122)
Occupation-working	39.0% (n = 78)
Prior information (yes)	48.0% (n = 96)

**Table 2. Changes in Anaesthesia Preference before and after Structured Preoperative Counseling (n = 200):** 

**Initial preference**	**Post-counseling spinal**	**Post-counseling general**	**Total**
Spinal	95	11	106
General	82	12	94
Total	177	23	200

McNemar test: *P* < 0.001. Values represent anaesthesia preference before and after counseling, not the final anaesthesia technique administered

**Table 3. Comparison of Patients According to Initial Anaesthesia Preference:** 

**Variable**	**Spinal (n = 106)**	**General (n = 94)**	***P*** **value**	**Effect size**
Age (years)	26.8±3.7	28.1±4.0	0.018	0.34 (Cohen’s d)
Gravida	2.2±0.9	2.4±1.1	0.210	0.20 (Cohen’s d)
Parity	1.0±0.7	1.2±0.9	0.095	0.25 (Cohen’s d)
Gestational age (weeks)	38.0±0.8	38.1±0.7	0.410	0.13 (Cohen’s d)
Higher education (%)	61.3%	46.8%	0.040	0.15 (φ)
Prior information (%)	55%	40%	0.041	0.15 (φ)
Previous spinal anaesthesia (%)	48%	22%	0.001	0.27 (φ)
Smoking (%)	10%	17%	0.120	0.10 (φ)
Comorbidity (%)	15%	19%	0.350	0.05 (φ)

Data are presented as mean ± standard deviation or percentage. Continuous variables were compared using Student’s t-test or the Mann-Whitney U test, as appropriate. Categorical variables were compared using the chi-square test or Fisher’s exact test. Effect sizes are presented as Cohen’s d for continuous variables and the phi coefficient (φ) for categorical variables

**Table 4. Multivariable Logistic Regression Analysis for Factors Associated with Spinal Anaesthesia Preference:** 

**Variable**	**OR**	**95% CI**	***P*** **value**
Age	0.94	0.89-0.99	0.021
Higher education	1.78	1.05-3.02	0.032
Prior information	1.65	1.02-2.68	0.041
Previous spinal anaesthesia	2.95	1.65-5.28	0.001
Smoking	0.72	0.38-1.35	0.310
Comorbidity	0.81	0.42-1.58	0.540

OR, odds ratio; CI, confidence interval
